# Refinement of lentiviral vector for improved RNA processing and reduced rates of self inactivation repair

**DOI:** 10.1186/1472-6750-9-86

**Published:** 2009-10-07

**Authors:** Rachel M Koldej, Donald S Anson

**Affiliations:** 1 SA Pathology, North Adelaide, South Australia, Australia; 2 Department of Paediatrics, The University of Adelaide, South Australia, 5005, Australia; 3 Current address : St Jude Children's Research Hospital, Department of Hematology, Mail Stop 341, 262 Danny Thomas Place, Memphis TN 38105, USA

## Abstract

**Background:**

Lentiviral gene therapy vectors are now finding clinical application. In order to fully exploit their potential it is important that vectors are made as efficient and as safe as possible. Accordingly, we have modified a previously reported vector to improve RNA processing, minimise Human Immunodeficiency Virus Type-1 (HIV-1) sequence content and reduce repair of the self inactivating (SIN) deletion.

**Results:**

HIV-1 sequence in the vector was reduced by substituting the polyadenylation signal with a heterologous signal. Mutation of splice donor sites was undertaken to prevent the majority of splicing within the vector genomic RNA. In addition, a number of other sequences within the vector were deleted. The combination of these modifications was able to significantly reduce the rates of both vector mobilisation and repair of the self inactivating deletion after two rounds of marker rescue.

**Conclusion:**

RNA processing can be improved by mutation of the major and minor HIV-1 splice donor sites in the vector. In addition the rate of vector mobilisation and repair of SIN vectors can be successfully reduced by careful vector design that reduces homology between the 5' and 3' long terminal repeats (LTRs) to a minimum.

## Background

Human immunodeficiency Virus Type 1 (HIV-1) derived gene therapy vectors are being used in the development of gene therapy treatments of many disorders [1-5], principally due to their ability to transduce dividing and non-dividing cells, resulting in long term stable expression of the desired transgene [6]. Also, compared to their oncoretroviral counterparts, once pseudotyped they are relatively easy to concentrate to high titres [7]. However, fears regarding the safety of HIV-1 derived vectors have persisted, especially due to the demonstrated oncogenic potential of other retroviral vectors.

Self Inactivating (SIN) vectors are designed to prevent the transfer of enhancer and promoter elements in the 5' long terminal repeat (LTR) of the vector to transduced cells [8]. These vectors contain a deletion in the 3' U3 sequence which is transferred to the 5' U3 sequence during reverse transcription, resulting in a provirus that contains no LTR derived enhancer or promoter elements [8], reducing the probability of proviral mediated gene activation. In SIN vectors, an internal heterologous promoter is used to express the transgene. As no full length RNA is produced by the integrated vector, the chances of inducing aberrant expression of nearby genes *via *3' LTR read-through, and vector mobilisation by other viruses infecting the cell [8,9], is decreased. SIN vectors also exhibit improved transgene expression [9], particle production and transduction efficiency [10] compared to their non-SIN counterparts. However, repair of the SIN deletion has been shown to occur at a measurable frequency [8], meaning these vectors do not entirely eliminate the possibility of Replication Competent Retrovirus production, vector mobilization or gene activation.

These issues are more than hypothetical. The treatment of X-linked severe combined immunodeficiency (X-SCID) with an oncoretroviral vector has demonstrated that the deregulation of gene(s), resulting from the integration of a viral genome near the gene(s) in question, can have serious consequences [11-13]. In approximately one quarter of the patients treated, the virus integrated near to an oncogene, most commonly LMO2, leading to deregulation of the oncogenic locus and a T cell Acute Lymphoblastic Leukaemia. Similarly, in the treatment of X-linked chronic granulomatous disease using an oncoretroviral vector, clonal populations have emerged in both patients treated thus far [14]. Whilst there is a difference in the integration site preference between oncoretroviral and lentiviral vectors [15-17], this does not exclude the possibility of a similar result occurring when HIV-1 derived gene therapy vectors are utilised, especially since repair of the SIN deletion can occur. This suggests that further development of lentiviral vectors are required to reduce safety concerns associated with the use of these vectors.

In this study, in order to improve the safety of the transfer vector, homology between the transfer vector and HIV-1, and between the vector LTRs, was reduced, resulting in decreased rates of SIN repair. In addition, a systematic analysis of the transfer vector sequence was undertaken to minimise the amount of HIV-1 sequence within the vector and to remove signals that adversely impact on the processing of the viral genomic RNA (gRNA). This analysis was able to prevent splicing of the gRNA, replace the 3' polyadenylation signal and reduce the size of the transfer vector.

## Results

### Splice site modifications

Splicing is used by HIV-1 to produce multiple mRNA transcripts encoding various proteins [18]. However, in the context of a HIV-1 derived gene transfer vector, splicing is unnecessary and can lead to a reduction in the amount of genomic RNA (gRNA) available for packaging. Therefore, we chose to mutate the HIV-1 splice donor site such that splicing no longer occurs.

**Figure 1:**
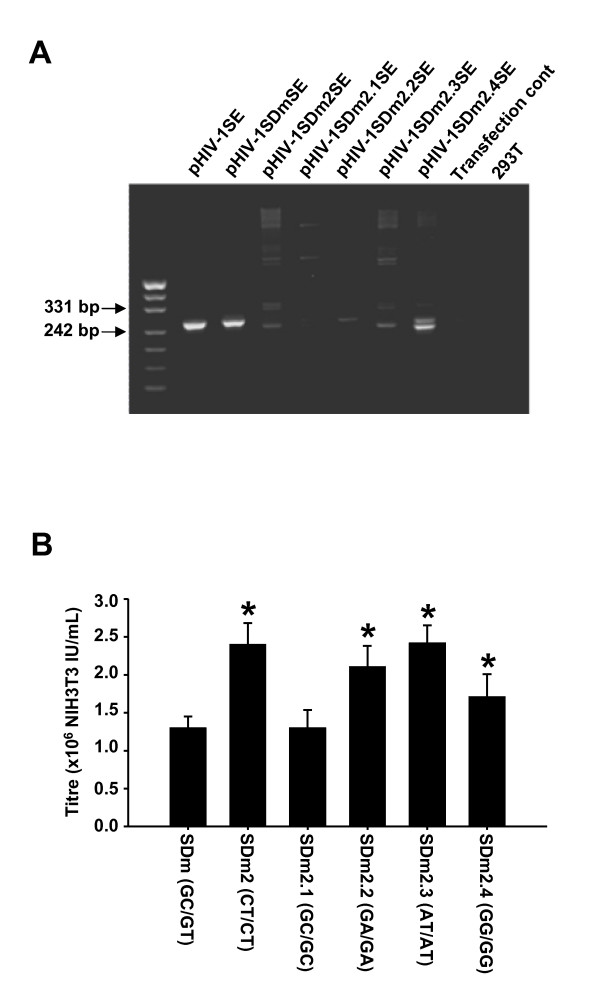
**Effect of Splice Site Mutation**. (A) Analysis of splicing in splice site mutation vectors. RNA from 293T cells transfected with the relevant vector, pcDNA3.1tat101ml and pHCMVrevmlwhv [37] was reverse transcribed and PCR was used to amplify spliced transcripts as described in Methods. The brackets indicate the mutation of the major and minor splice donor sites present in each vector. SDm, pHIV-1SDmSE; SDm2, pHIV-1SDm2SE; SDm2.1, pHIV-1SDm2.1SE; SDm2.2, pHIV-1SDm2.2SE; SDm2.3, pHIV-12.3SE; SDm2.4, pHIV-1SDm2.4SE. (B) Vector titres using 0.125 ug vector per well (see Materials and methods for details). *All SDm2 variants, with the exception of SDm2.1, had significantly higher titres than the parental vector, SDm (ANOVA/Holm-Sidak, p < 0.01). In addition, SDm2, SDm2.2, SDm2.3 and SDm2.4 all had significantly higher titres than SDm2.1 (ANOVA/Holm-Sidak, p < 0.01). All results are presented as Mean ± SD (n = 6).

We have previously shown that in our vector pHIV-1SE, that splicing occurs between the HIV-1 major splice donor and cryptic splice acceptor sites in the SV40 promoter sequence [19]. Mutation of the major splice donor site from GT to CG (pHIV-1SDmSE) reduced but did not abrogate splicing (Figure 1a, lanes 1 and 2). DNA sequencing indicated that the residual splicing originated at the minor splice donor site 2 bases 3' of the major splice donor site. Therefore, vectors which contained mutations to both the major and minor splice donor sites were constructed. Five different mutations were evaluated, in these both splice donor sites were mutated from GT to (i) CT (pHIV-1SDm2SE); (ii) GA (pHIV-1SDm2.1SE); (iii) GG (pHIV-1SDm2.2SE); (iv) AT (pHIV-1SDm2.3SE) or (v) GC (pHIV-1SDm2.4SE).

Qualitative RT-PCR showed that all vectors except pHIV-1SDm2.4SE produce less spliced RNA compared to pHIV-1SDmSE (Figure 1a). The titres of the vectors pHIV-1SDm2SE, pHIV-1SDm2.2SE and pHIV-1SDm2.3SE were equal and approximately 2-fold higher than pHIV-1SDmSE (p < 0.01, ANOVA/Holm-Sidak) whilst pHIV-1SDm2.1SE gave a titre equal to pHIV-1SDmSE (Figure 1b). pHIV-1SDm2SE was chosen for further studies as it offered high titres in combination with very low levels of residual RNA splicing.

### 3'U3 modifications

In order to reduce homology between the 5 and 3' LTRs the effect of replacing the HIV-1 polyadenylation signal with either the SV40 (Genbank accession number NC001669, bp 2606 to 2700) or bGH (Genbank accession number M57764, bp 2413 to 2465) polyadenylation signals was investigated. For the bGH vector the polyadenylation signal was inserted after the *Afl*II site in the R sequence so that it replaced the last 25 bp of R and all of U5. For the SV40 vector the polyadenylation signal was inserted 8 bp after the *Sac*I site in the R sequence so that it replaced the last 51 bp of R and all of U5. In both constructs all of U3, with the exception of the 19 bp *att *sequence was deleted, as was all extraneous sequence between the end of the EYFP coding sequence and the start of the PPT (we have previously shown that deletion of the latter sequence has no effect on vector performance [20]). The resulting constructs were designated pHIV1SDm2SESV*att*19 and pHIV1SDm2SEbGH*att*19.

**Figure 2:**
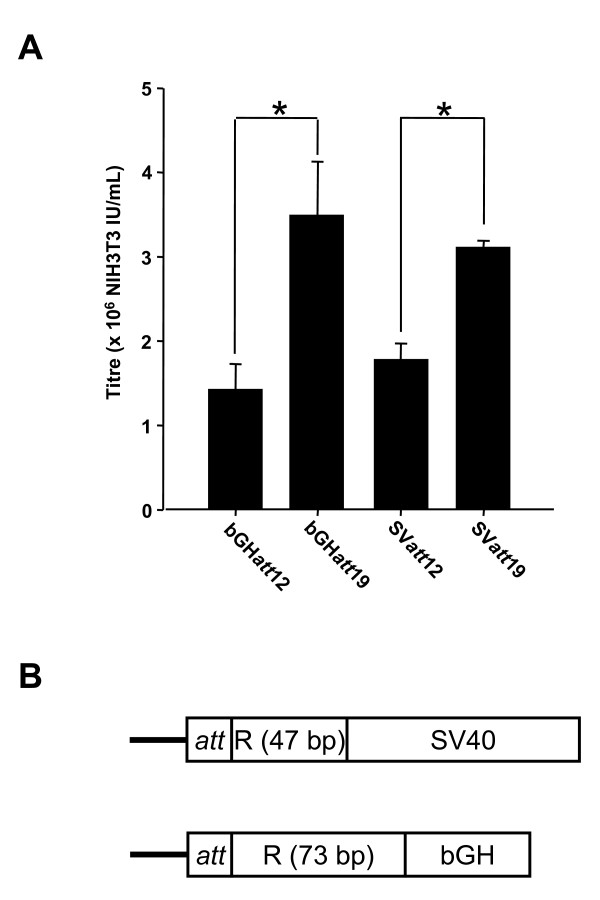
**Effect of 3'U3 alterations on virus titre**. (A) Titres of vectors containing either the SV40 or bGH polyadenylation sites, and either a 12 or 19 bp *att *sequence were compared using 0.125 ug vector per well (n = 3). bGH*att*12, pHIV-1SDm2SEbGH*att*12; bGH*att*19, pHIV-1SDm2SEbGH*att*19; SV*att*12, pHIV-1SDm2SESV*att*12; SV*att*19, pHIV-1SDm2SESV*att*19. * = P < 0.05 ANOVA on Ranks. All results are presented as Mean ± SD. (B) Schematic of 3' LTR structures. In both cases R is truncated at the 3' end, *att *is 12 or 19 bp in length. SV40, SV40 early polyadenylation signal; bGH, bovine growth hormone gene polyadenylation signal (see Results for details).

When tested for virus titre using amounts of vector DNA for transfection (0.125 μg/well) that were limiting for virus production, there was no significant difference in titre between the vectors (data not shown). Reducing the *att *sequence to 12 bp in length resulted in significantly lower titres (Figure 2). From this analysis the bGH*att*19 and SV40*att*19 constructs appeared equivalent to the pHIV-1SDm2SE vector, with the apparent advantage that they contain less HIV-1 sequence than the latter.

### Combination gag and 3'U3 deletion vectors

**Figure 3:**
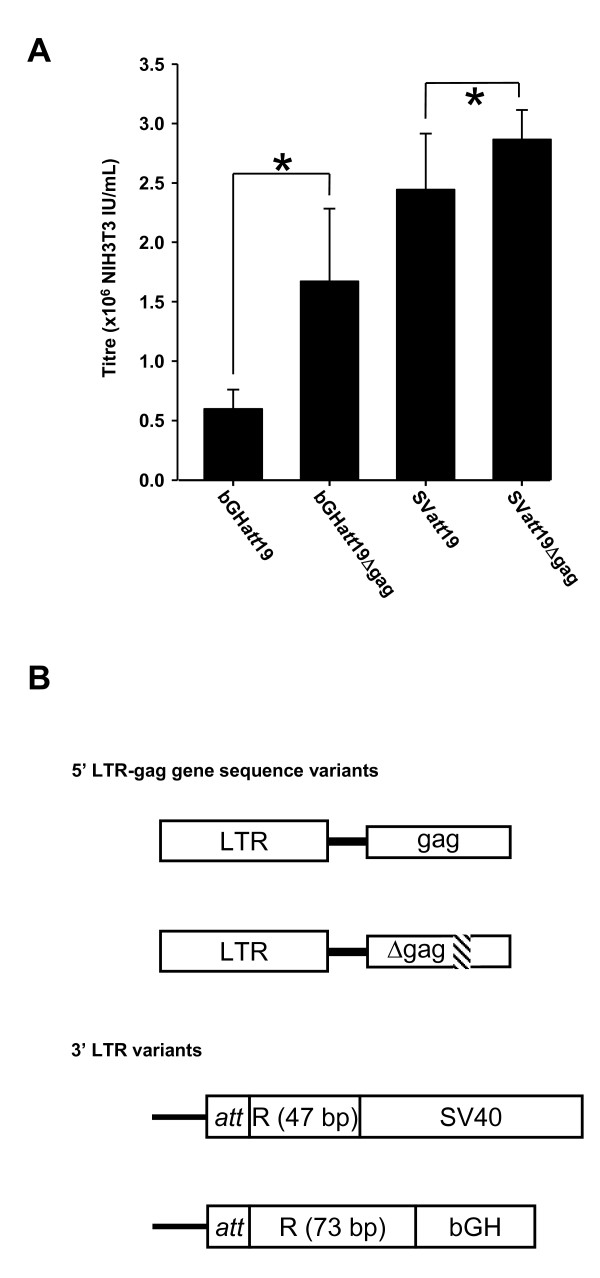
**Effect of combination *gag *and 3'U3 modifications on virus titre**. (A) Vectors containing both the Δ*gag *and 3'U3 deletions were compared for virus titre using 0.125 ug vector per well. bGH*att*19, pHIV1SDm2SEbGH*att*19; bGH*att*19Δgag, pHIV1SDm2SEbGH*att*19Δgag; SV*att*19, pHIV1SDm2SESV*att*19; SV*att*19Δgag, pHIV1SDm2SESV*att*19Δgag (pRK1). * = P < 0.001, ANOVA with Holm-Sidak multiple comparison. All results are presented as Mean ± SD (n = 6). (B) Schematic of 5' LTR and *gag *gene sequence and 3' LTR variants present in the constructs tested. The region of the *gag *gene deleted in the "Δgag" constructs is indicated by cross hatching. All constructs contain a 19 bp *att *sequence in the 3' LTR and the R sequence is truncated at the 3' end.

Deletion mapping demonstrated that bases 325-400 of the *gag *gene sequence present in our original vector could be deleted without significantly affecting virus titre (data not shown). This deletion (Δgag) was transferred into pHIV-1SDm2SEbGH*att*19 and pHIV-1SDm2SESV*att*19. In both cases, the addition of this deletion increased the resulting virus titre compared to the parental vectors (Figure 3). The vector pHIV-1SDmSE2SV40*att*19Δgag was chosen for further analysis as it exhibited the highest titre. For simplicity, this construct was renamed pRK1.

### 5'U3 modifications

**Figure 4:**
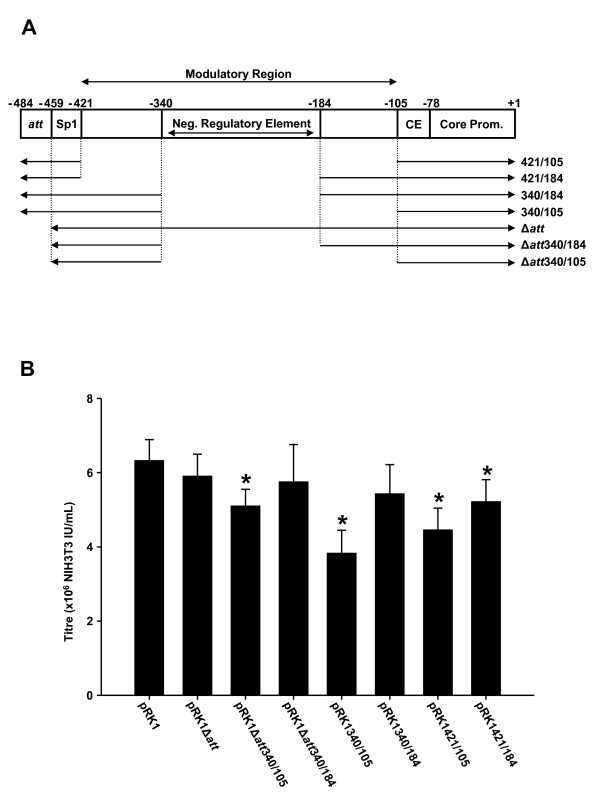
**Effect of 5'U3 deletions on virus titre**. (A) Schematic showing the deletions to the modulatory region of 5'U3 that were analysed. *att*, integration signal; Sp1, Sp1 binding site; CE, core enhancer. (B) Vector titres using 1 ug vector per well. * = P < 0.05 vs. pRK1, ANOVA on Ranks with Dunns multiple comparison. All results are presented as Mean ± SD (n = 6).

The HIV-1 5'U3 contains a modulatory region between -421 and -105, included in which is a negative regulatory element between -340 and -184 [21,22], as well as core enhancer and promoter sequences. Deletion mapping (Figure 4a) was used to determine if the modulatory and negative regulatory elements are required in the context of a HIV-1 derived gene transfer vector. In addition, deletion of the 5'*att *sequence was assessed as this sequence would appear to be redundant given that the *att *sequence is duplicated from the 3' LTR upon reverse transcription. The titres of pRK1Δ*att*, pRK1Δ*att*340/184 and pRK1340/184 were not significantly different to that of pRK1 (Figure 4b) while all the other deletions lead to a significant decrease in virus titre (p < 0.05, ANOVA on ranks with Dunns Multiple Comparisons).

### 5' polyadenylation signal

**Figure 5:**
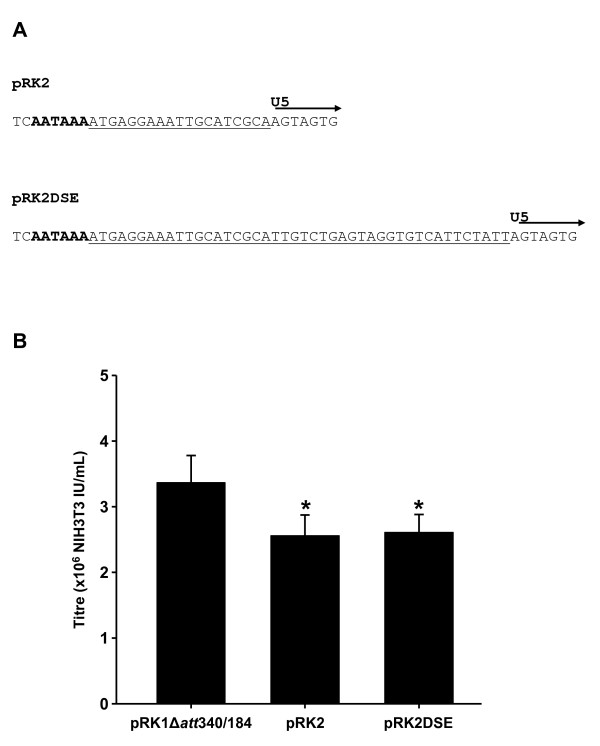
**The effect of insertion of the bGH polyadenylation signal into the 5' LTR**. The bGH polyadenylation signal was inserted into the 5' LTR with (pRK2DSE), or without (pRK2), the downstream regulatory element and vector titres assessed. (A) Sequence of insertions made. The AATAAA motif is shown bold and the bGH sequence is underlined. (B) Vector titres using 1 ug vector per well. * = P < 0.05 vs pRK1Δ*att*340/184 ANOVA on Ranks with Dunns Multiple Comparison All results are presented as Mean ± SD (n = 6).

Retroviral polyadenylation signals are thought to be inherently leaky as they must allow read-through of the 5' LTR so that the genome can be efficiently transcribed [21]. While substitution of the HIV-1 polyadenylation signal with the SV40 polyadenylation signal in the 3' LTR of the vector results in the SV40 polyadenylation signal being utilised during virus production, during reverse transcription it is 5'R/U5 which acts as a template for the cDNA, resulting in the HIV-1 polyadenylation signal being used in the resulting provirus. Therefore, to change the polyadenylation signal used in transduced cells, the sequence of the polyadenylation signal in the 5' LTR must be replaced. To this end the bGH polyadenylation signal was inserted into the 5'LTR of pRK1Δ*att*340/184. As the bGH polyadenylation signal contains a downstream sequence element (DSE) that increases the accuracy, but not efficiency of polyadenylation, two variants were created, one without (pRK1Δ*att*340/184bGH, renamed pRK2), and one with (pRK1Δ*att*340/184bGHDSE renamed pRK2DSE) the bGH polyadenylation signal DSE (Genbank M57764 bases 2422-2440 and 2422-2465 respectively) inserted after the AATAAA motif in R, so as to replace the last 19 bp of R (Figure 5a). In both these constructs a small decrease in virus titre was seen (Figure 5b) compared with the parental vector (P < 0.05 ANOVA on Ranks with Dunns Multiple Comparison).

**Figure 6:**
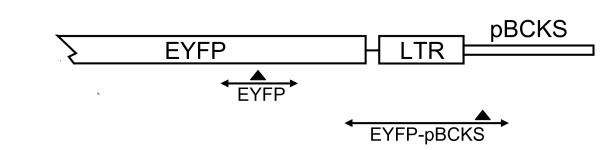
**Assay for transcriptional read-through of the 3' LTR**. Arrows indicate the real time PCR product and the solid triangle the position of the TaqMan probe. EYFP, enhanced yellow fluorescent protein; LTR, 3' long terminal repeat sequence.

We then assessed whether the use of the bGH polyadenylation signal in the provirus resulted in reduced transcriptional read-through compared with the HIV-1 signal. To allow this, vectors which had the structure of the 3' LTR post reverse transcription were created for pRK2 and pRK2DSE giving the vectors pRK2prt and pRK2DSEprt. The vector pHIV-1SDmSE was also analysed for read-through. However, as the structure of the 3' LTR pre and post reverse transcription in pHIV-1SDmSE is unchanged, this vector could be tested without modification. Each vector construct was transfected into 293T cells along with constructs expressing Tat and Rev and the resulting mRNA isolated and reverse transcribed. The total amount of vector transcripts, and the amount that is contiguous with sequences 3' of the polyadenylation signal, were then determined to allow the frequency of polyadenylation signal read-through to be measured. This was done using two real time PCR assays; one that detects the *eyfp *sequence present in the vector, and one that detects sequence spanning from *eyfp *into the *pBCKS *cloning vector (i.e. read-through transcript) (Figure 6).

The results of this assay (Table 1) indicated that the post reverse transcription 3' LTRs from both pRK2 and pRK2DSE actually exhibit increased levels of polyadenylation signal read-through compared to pHIV-1SDmSE. Therefore, these vectors were not considered further and the vector pRK1Δ*att*340/184 was chosen for further assessment.

**Table 1:** Rate of polyadenylation read-through (n = 3)

**Vector**	**Frequency of Read-through**^a^
pHIV-1SDmSE	9.05 ± 0.93 × 10^-3^
pRK2	8.70 ± 2.41 × 10^-3^
pRK2prt	8.57 ± 3.50 × 10^-2^
pRK2DSE	1.35 ± 0.41 × 10^-2^
pRK2DSEprt	8.72 ± 1.42 × 10^-2b^

^a^Real time PCR was used to determine the frequency of polyadenylation signal read-through in 293T cells transduced with the vector, pcDNA3.1Tat101ml and pHCMVrevnlwhvpre. Results are Mean ± SD.

^b^P < 0.05 vs pHIV-1SDmSE, pRK2 and pRK2DSE, t-test

### Detailed Comparison of pRK1 and pRK1Δatt340/184 with pHIV-1SDmSE

**Figure 7:**
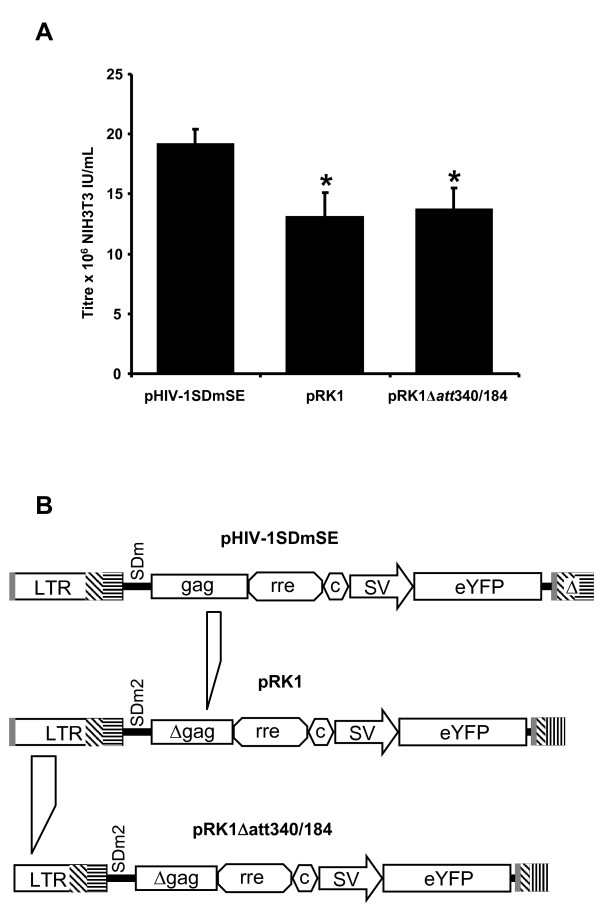
**Titre comparison of pHIV-1SDmSE, pRK1 and pRK1Δ*att*340/184**. (A) The titre of pHIV-1SDmSE, pRK1 and pRK1Δ*att*340/184 using 1 ug vector per well. *P < 0.01 versus pHIV-1SDmSE ANOVA All results are presented as Mean ± SD (n = 18). (B) Schematic diagrams of the vectors assessed. Grey vertical bar represent the *att *sequence; LTR, long terminal repeat; diagonal cross hatching, R sequence; horizontal hatching, U5 sequence; SDm(2), mutagenised splice donor site(s); gag, *gag *gene sequence; rre, Rev response element; c, central polypurine tract; SV, SV40 early promoter; eYFP, enhanced yellow fluorescent protein coding sequence, Δ, SIN LTR; vertical hatching, SV40 polyadenylation signal.

The pRK1 and pRK1Δ*att*340/184 vectors were directly compared with the parental plasmid pHIV-1SDmSE in a number of assays to see if the changes incorporated into pRK1 and pRK1Δ*att*340/184 resulted in an improved vector. Assessment of titre showed that both the modified vectors had a small (approximately 1/4) but significant (p < 0.01, ANOVA) reduction in titre compared with pHIV-1SDmSE (Figure 7). We note that the titres reported in Figure 7 are significantly higher than those reported in Figure 4. Our best explanation for this is that the experiments were done over 1 year apart, and used different batches of transfection reagents and cells cultured from different stocks.

The propensity for pRK1neo and pRK1Δ*att*340/184neo to repair the SIN deletion in the 3'LTR was then assessed using a marker rescue assay. For this analysis vectors carrying the neomycin resistance gene were used in order to be able to detect unitary events. In this assay, cells which have been transduced by each virus are transfected with plasmids encoding proteins necessary for virus production (i.e. Tat, Rev, GagPol and VSV-G). If the deletion is repaired, the cell will contain full length genomic transcripts which can be packaged, resulting in virus production. However, it is also possible that transcription from a nearby upstream active promoter will result in a packagable message. Therefore, to selectively amplify the events where the LTR has been repaired, the virus rescued in the first instance was used to transduce cells and a second round of rescue performed. The vectors pHIV-1SDmSneoLTR (a vector containing unmodified and intact 5' and 3' LTRs), and pHIV-1SDmSneo were utilised as assay controls.

**Figure 8:**
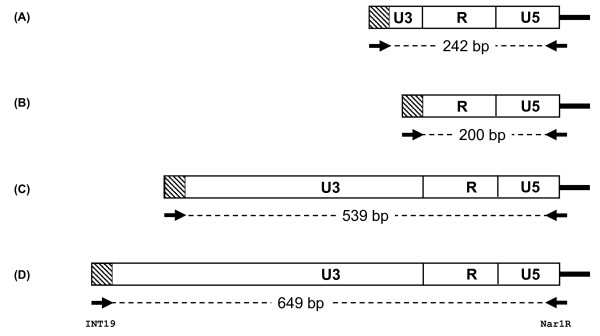
**Structure of proviral 5' LTRs after reverse transcription and integration**. The predicted structures of the 5' LTRs, both SIN and repaired, for the pHIV-1SDmSneo, pRK1neo and pRK1Δ*att*340/184neo vectors, and the corresponding size of the expected PCR product from analysis of genomic DNA with the primers int19 and Nar1R are shown. (A) pHIV-1SDmSneo SIN LTR; (B) pRK1neo/pRK1Δ*att*340/184neo SIN LTR; (C) pRK1Δ*att*340/184neo repaired LTR; (D) pHIV-1SDmSneo/pRK1neo repaired LTR. See Methods for details and Table 2 for results.

**Figure 9:**
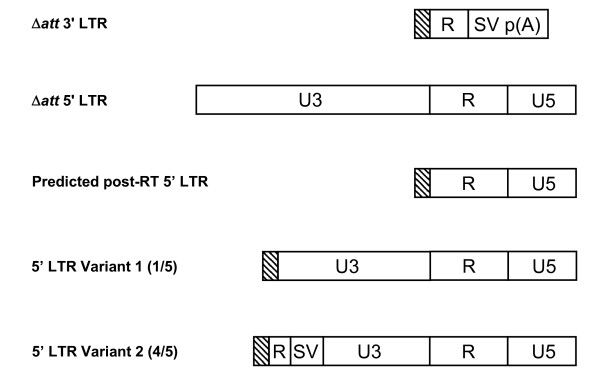
**Sequence analysis of intermediate size PCR products from the pRK1Δ*att*340/184neo rescue assay**. DNA was prepared from colonies arising from the second round of the pRK1Δ*att*340/184neo provirus rescue assay and analysed by PCR as described in Methods. PCR products that were intermediate in size to the SIN LTR and the repaired LTR were sequenced and showed partial repair of the U3 sequence, with the sequence truncated at the 5' end. Four of these clones also contained partial duplications of the R/SV40 polyadenylation sequence from the 3' LTR. Δatt 3' LTR, structure of the 3' LTR from pRK1Δ*att*340/184neo; Δatt 5' LTR, structure of the 5' LTR from pRK1Δ*att*340/184neo; predicted post-RT 5' LTR, structure of the pRK1Δ*att*340/184neo proviral 5' LTR after reverse transcription and integration; 5' LTR variant 1 and 2, structure of sequenced PCR products for which the PCR product did not correspond to the size predicted for the SIN or the repaired LTR. Of the 5 PCR products sequenced, 4 corresponded to variant 2.

After 2 rounds of rescue, pHIV-1SDmSneo was rescued 2-fold more efficiently than pRK1neo (P = 0.129, t-test) and 8.5-fold more efficiently than pRK1Δ*att*340/184neo (P < 0.03, t-test) (Table 2), meaning pRK1Δ*att*340/184neo was rescued 4.4-fold less efficiently than pRK1neo (P < 0.005, t-test). To further examine the differences between these vectors, individual neomycin resistant colonies from the virus assay of the round 2 rescued virus were isolated using cloning rings and the genomic DNA analysed for the structure of the 5' LTR using PCR. The 5' primer for this PCR was designed to bind to the *att *sequence in the 5' LTR and the 3' primer to the primer binding site. A fully repaired 5' LTR will give a PCR product of 649 (pHIV-1SDmSneo and pRK1neo) or 539 (pRK1Δ*att*340/184neo) bases, whilst a non-repaired SIN 5' LTR will result in a PCR product of 244 (pHIV-1SDmSneo), or 200 (pRK1neo and pRK1Δ*att*340/184neo) bases (Figure 8). All colonies examined from pHIV-1SDmSneo and pHIV-1SDmSneoLTR exhibited fully repaired LTRs. In contrast 18% (4/22) of the colonies from pRK1neo that yielded PCR products, and 32% (4/11) of the colonies from pRK1Δ*att*340/184neo that yielded PCR products, were indicative of non-repaired SIN LTRs, demonstrating a significant improvement in the maintenance of the SIN phenotype in pRK1Δ*att*340/184neo (Table 2) (pHIV1SDmSneo v pRK1Δ*att*340/184neo P < 0.002, Fisher's exact Chi squared) but not in pRK1neo. The corresponding numbers for fully repaired LTRs were 59% (13/22) and 17% (2/11), respectively, meaning the rate of fully repaired LTRs was significantly reduced in pRK1Δ*att*340/184neo compared to both pRK1neo and pHIV-1SDmSneo (Fisher's exact Chi squared, p < 0.05 and p < 0.0001, respectively). Both pRK1neo (22% of colonies yielding PCR products) and pRK1Δ*att*340/184neo (42% of colonies yielding PCR products) also gave some PCR products of a size intermediate between the SIN LTR and the fully repaired LTR. Sequence analysis of intermediate size PCR products from pRK1Δ*att*340/184neo showed partial repair of the U3 sequence, with the sequence truncated at the 5' end. Of the 5 clones of this type that were successfully analysed, 4 also contained partial duplications of the R/SV40 polyadenylation sequence from the 3' LTR (Figure 9). In addition, while all colonies derived from pHIV-1SDmSneo and pHIV-1SDmSneoLTR yielded PCR products, 1 out of 23 colonies from pRK1neo, and 8 of 20 colonies from pRK1Δ*att*340/184neo, did not yield a PCR product, suggesting that at least one of the PCR primer sequences was missing.

**Table 2:** Marker rescue assay (n = 3)

**Vector**	**IU recovered/IU added^a^**		**Frequency of SIN 5' LTR**^d^	**Frequency of complete 5' LTR**^e^
	**Round 1^b^**	**Round 2^c^**		
pHIV-1SDmSneo	7.79 ± 0.62 × 10^-5^	0.21 ± 0.09	0% (0/17)	100% (17/17)
pHIV1SDmSneoLTR	0.71 ± 0.12	0.22 ± 0.40	0% (0/15)	100% (15/15)
pRK1neo	1.14 ± 0.47 × 10^-4^	0.11 ± 0.02	18% (4/22)	59% (13/22)
pRK1Δ*att*340/184neo	5.56 ± 1.33 × 10^-5^	2.52 ± 1.73 × 10^-2^	32% (4/11)	17% (2/11)

^a^Each vector was used to transfect 293T cells and subsequently rescued by transfection with pCMVΔRnr and pHCMV-g. The virus rescued in round 1 was used in a second round of transfection/rescue. Mean ± SD. ^b^The titre of rescued virus as determined on A549 cells and normalised to the amount of virus initially added. ^c^The titre of the virus rescued in the second transfection and normalised to the amount of virus rescued in round 1. ^d^Individual neomycin resistant colonies from the second round titre assay were analyzed via PCR for the structure of the 5'LTR (see also Figure 8), pRK1Δ*att*340/184neo significantly different to pHIV-1SDmSneo (Fisher's exact Chi squared, p < 0.002). ^e^Repair refers only to LTRs having a complete U3 sequence as determined from PCR analysis (pRK1Δ*att*340/184neo significantly different to pHIV1SDmSneo (p < 0.0001) and pRK1neo p < 0.05, Fisher's exact Chi squared). For pRK1neo 5 colonies produced PCR products of intermediate size and one colony out of 23 did not yield a PCR product. For pRK1Δ*att*340/184neo 5 colonies produced PCR products of intermediate size (see also Figure 9) and 8/20 colonies did not yield a PCR product. Colonies not producing PCR products were not scored in the results shown above.

## Discussion

While the broad design features for a successful lentiviral vector have been apparent for some time, we believe that refinement of vector design can lead to significant improvements in vector performance. As the issue of the production of replication competent virus appears to have already been solved in third generation lentiviral vector systems, in this study we have focussed on improving our vector in two ways (i) by improving RNA processing by minimising splicing and (ii) reducing the propensity for SIN repair and vector mobilisation, while maintaining vector titre, a principle determinate of vector utility.

In our analyses we generally found that intra-experiment variation was much less than the variation between experiments, and that the latter was greatly reduced if the experiment was repeated relatively contemporarily. In all cases, the observed relative differences between vectors were highly reproducible, even if the absolute titres were less so.

Splicing has an important role to play in the HIV-1 lifecycle. By alternately splicing the 9 kb genome, mRNA encoding multiple proteins can be produced [10]. However, this process is irrelevant in the context of a HIV-1 gene transfer vector, and can result in a decrease in the amount of full length gRNA available for packaging. Mutation of the cryptic splice acceptor sites in our vector had previously been attempted but was unable to reduce the amount of splicing as it resulted in other cryptic splice acceptor sites being used (D. Anson, unpublished). Therefore, we chose to mutate both the major and minor HIV-1 splice donor sites as cryptic splice donor sites appear to be less common. This is presumably due to the more rigid consensus sequence for splice donor sites (AG**GU**RUAGU) than splice acceptor sites (N**AG**G) [23].

Mutation of the HIV-1 major splice donor site has been previously attempted [24]. The site was mutated from GT to CA or GG. These modifications were only able to prevent splicing to a limited extent due to the activity of the HIV-1 minor splice donor site. In the present study splicing could only be reduced by simultaneously mutating both the major and minor splice sites. However, not all mutations were equally effective. Closer examination revealed the mutations which contained a purine as the second base of the mutation lead to a significant increase in virus titre and a reduced amount of splicing. As the splice donor sites are very close to the highly structured packaging signal [21], this requirement for a purine as the second base of the splice donor sites may be due to the effect of the mutation on the secondary structure of the mRNA. The residual amount of splicing seen is most likely due to the use of cryptic splice donor sites [25]. Presumably, the mutation of the major and minor splice donor sites would be effective in reducing splicing to any splice acceptor in the vector, and therefore provides a general method for reducing unwanted vector splicing.

Modifications to the 3' LTR were able to reduce the amount of HIV-1 sequence within the vector. The HIV-1 polyadenylation signal was successfully substituted with the bGH or SV40 polyadenylation signal. In addition all of U3, except the *att *sequence, was successfully deleted. Due to conflicting reports regarding the exact size of *att *[26,27], both 12 and 19 bp sequences were assessed. In both vectors, the use of the 19 bp *att *sequence resulted in higher virus titres.

Deletion mapping was used to identify a 75 bp sequence in *gag *which was able to be removed from the transfer vector without significantly affecting virus titre. This region can be mapped to a *Cis *Repressive Sequence (CRS) within *gag*. These CRS are responsible for the retention of the unspliced HIV-1 mRNA in the nucleus prior to production of Rev [28,29]. There are many CRS sequences within *gag*, 4 of which are within the *gag *coding sequence in the transfer vector. The deletion described here was able to remove one of these regions. The removal of the others was not possible as their sequences are necessary for efficient virus production. However, mutation of these CRS elements is possible and has been shown to lead to Rev independent Gag expression in HIV-1 [29,30]. Therefore, it may be beneficial to examine these mutations in the context of the transfer vector to determine their effect on gRNA levels and virus production. However, whether this would result in a Rev independent vector is unclear. The CRS elements direct the mRNA to distinct intranuclear locations [31] allowing the mRNA to bind to Rev and exit the nucleus along the correct pathway [32]. By deleting them, the mRNA may not exit the nucleus along the correct pathway, which may have repercussions for the viral assembly process and hence titre.

Within the field of lentiviral gene therapy vectors, most groups have chosen to replace the 5' LTR with a constitutive promoter (usually CMV) to allow Tat independent transcription [33,34]. This reduces the number of plasmids required for the production of the vector and also reduces the amount of HIV-1 sequence within the production system as a whole, and is assumed to reduce the probability of RCL being produced and of SIN deletion repair occurring. However, given that none of the third generation vector systems generate measurable RCL, and SIN repair is not usually assessed, the degree to which this approach to vector design improves safety is hard to assess. We note that the use of a heterologous promoter to replace U3 in the 5' LTR also removes a level of regulation from the vector, and that using the HIV-1 5' LTR requires that an additional recombination step must occur (i.e. the Tat coding sequence must be present) for the formation of a replication competent lentivirus, or for active transcription of a provirus *via *a repaired SIN deletion (see below) to take place. In our view, this means that the retention of the HIV-1 5' LTR in the vector may be preferable. Clearly, these two approaches to vector design need to be directly compared with respect to (i) the likelihood of generating RCL, and (ii) the rate of SIN repair, to resolve which is preferable. Given that measurable RCL is not generated by advanced lentiviral vector systems, a surrogate assay, such as quantitative assessment of recombination intermediate(s) that could lead to the generation of RCL may be a more appropriate assay to use.

The 5' LTR contains many regulatory elements, most of which are within U3 [21,22]. Included in this sequence is the modulatory region which, whilst important for wild type HIV-1, may not be required in the context of a HIV-1 derived gene transfer vector. Deletion mapping established that the negative regulatory element (from -340 bp to -184 bp) within the modulatory region was not required. In addition, the *att *sequence in the 5' LTR could also be deleted as this sequence is also not required in the context of a HIV-1 derived gene transfer vector (the *att *sequence in the 3' LTR being copied to the 5' LTR during reverse transcription).

We also successfully showed that a heterologous polyadenylation signal could be incorporated into the 5' LTR, and hence the provirus. The use of the bGH polyadenylation signal, either with or without the downstream sequence element, to replace the HIV-1 polyadenylation signal in the 5' LTR was successful in vectors containing an SV40 polyadenylation signal in the 3' LTR. However, when tested, it was shown that insertion of the bGH polyadenylation signal resulted in an increase in polyadenylation signal read-through in the provirus. Therefore, the use of a heterologous polyadenylation signal in the 5' LTR, while technically possible, does not appear to offer any advantages in polyadenylation efficiency over the HIV-1 signal, and so was not used in our final vectors. A similar vector in which the SV40 polyadenylation signal was incorporated into the 5' LTR was unsuccessful, with titre being reduced to very low levels (data not shown). These results may reflect the inherent conflict between the necessity for poly(A) read-through in the 5' LTR and the wish for efficient polyadenylation in the 3' LTR.

Detailed comparison of pHIV-1SDmSneo, pRK1neo and pRK1Δ*att*340/184neo showed that there was a significant difference in the ability of the viral genome to be rescued upon expression of packaging functions. The use of two rounds of rescue in our assay was vital in showing this difference. Previous attempts to measure the rate of SIN repair have analysed repair after a single round of transduction [8,35]. However, this does not distinguish between repair of the promoter and transcription from cryptic promoters upstream of the LTR [36]. The transcripts from cryptic promoters do not contain a repaired LTR, and therefore are unlikely to produce virus capable of undergoing a further round of replication. The power of our 2-stage assay is exemplified by the fact that with pHIV-1SDmSneo, 100% of first round colonies examined for LTR status were SIN, (data not shown) while 100% of second round colonies were repaired, demonstrating extremely strong selection for LTR repair in the assay. Interestingly, it was only when homology between the U3 regions had been completely eliminated that the rate of SIN repair was significantly reduced. Even when homology was reduced to just 19 bases in the U3 regions, as in pRK1neo, the rate of rescue was only reduced two-fold, which was not significantly different to pHIV-1SDmSneo. Similarly, while the percentage of complete repair of the SIN deletion in secondary isolates was reduced, this was only to 59% of the rate in pHIV-1SDmSneo and again was not significantly different from pHIV-1SDmSneo. In fact, repair still occurred in the absence of any homology between the U3 regions, as in pRK1Δ*att*340/184neo. However, in this instance there was a significant reduction in both the rate of rescue, and the percentage of repaired LTRs compared to pHIV-1SDmSneo and to pRK1neo, and a significant increase in the percentage of SIN LTRs compared to pHIV-1SDmSneo. Sequence analysis of colonies from the pRK1Δ*att*340/184neo colonies showed that the PCR products of the size predicted for repaired LTRs with a complete U3 sequence or retaining the full SIN deletion (Figure 8) were indeed of the expected structure. In addition, partially repaired LTR sequences were also seen which contain a U3 sequence truncated at the 5' end, several of these LTRs also contained partial repeats of the R and SV40 sequences (Figure 9). These characteristics (reduced rescue and reduced SIN deletion repair) of pRK1Δ*att*340/184neo should reduce the probability of vector mobilisation and of a replication competent virus being produced. Whilst the mechanism of repair is unclear, one possibility is that repair may occur during the reverse transcription process. This in turn would suggest that the use of stable cell lines to produce HIV-1 derived vectors may not eliminate the issue of SIN repair.

This finding also has important implications for HIV-1 vector design. A number of groups have chosen to replace the HIV-1 5'U3 sequence with a CMV promoter [33,34]. If similar SIN deletion repair (i.e. repair not requiring sequence homology between the U3 region of the 5' and 3' LTRs) occurred in these vectors, transduced cells would contain a hybrid LTR with strong CMV derived enhancer elements, increasing the potential for both virus rescue/mobilisation and enhancer mediated gene activation [12]. By using the HIV-1 promoter to drive transcription of the viral genome during production, even if recombination does occur, the provirus should remain transcriptionally silent, reducing the probability of vector mobilisation, although the issue of enhancer mediated gene activation would remain. In our view further investigation is warranted to determine the mechanisms of SIN deletion repair in vectors with non-homologous U3 sequences, and to determine if such repair occurs in vectors with heterologous 5' LTR (U3) promoters. If SIN deletion repair does occur in vectors with heterologous 5' LTR (U3) promoters then the suitability of these vectors for clinical application may need to be re-evaluated.

## Conclusion

Our careful systematic analysis of the vector sequence has improved RNA processing and more importantly has allowed a reduction in the amount of HIV-1 sequence within the vector resulting in significant improvements in the rate of repair of the SIN deletion.

## Methods

### Cell Culture

All cell lines were maintained at 37°C/5% CO_2 _with regular subculturing using a trypsin/EDTA solution (SAFC Biosciences) as required. 293T (ATCC CRL 11268) and A549 (ATCC CCL 185) cells were maintained in DMEM/5% (v/v) FCS containing 4.5 g/L glucose and 4 mM glutamine (SAFC Biosciences). NIH3T3 (ATCC CRL 1658) cells were maintained in DMEM/10% (v/v) FCS containing 4.5 g/L glucose and 4 mM glutamine (SAFC Biosciences).

### Plasmid Construction

Plasmids were created utilising standard DNA cloning methods. The parental vector pHIV-1SDmSE has been previously described [37].

### Virus Production

293T cells were plated 24 hours prior to transfection at a density of 0.9 × 10^6 ^cells in 2 mL DMEM/5% (v/v) FCS per well of a 6 well plate. The cells were then transduced by calcium phosphate co-precipitation as previously described [37]. Each well was transfected with 1 or 0.125 μg of the appropriate transfer vector, 1.5 μg pCMVΔRnr [2] and 0.5 μg of pCMV-g [38]. Eight hours post transfection the media was exchanged for fresh DMEM/5% (v/v) FCS and the virus harvested 48 hours post transfection.

### Virus Titre Assays

#### EYFP

Viral titres of EYFP lentiviruses were determined on NIH3T3 cells using FACScan analysis. NIH3T3 cells were plated at a density of 0.25 × 10^6 ^cells in 0.5 mL medium per well of a 24 well plate. Three hours later the media was changed for medium supplemented with 4 μg per mL polybrene (Sigma) and 50 μg per mL Gentamycin (Sigma) and an appropriate volume of virus was added such that 5-20% of the cells would be positive for EYFP expression. The media was exchanged for growth medium after 24 hours and incubation continued for a further 24 hours. The cells were then split 1:2 and cultured for another 24 hours. The cells were then harvested, the live cell population was gated by side and forward scatter and EYFP expression analysed through the FITC channel on a Becton Dickinson FACScan Machine. The results were analysed using Cell-quest software (version 3.0.1f, Becton Dickinson).

#### Neomycin Resistance

A549 cells were plated at a density of 0.5 × 10^6 ^cells/well in a 12 well plate (1 mL/well). After 3 hours incubation, the media was changed for DMEM/5% (v/v) FCS/4 μg per mL polybrene (Sigma) and an appropriate amount of virus was added (aiming for between 10 and 100 IU/well). The assay was incubated at 37°C/5% CO_2 _for 24 hours before the cells in each well were transferred to a 100 mm dish containing 12 mL DMEM/5% (v/v) FCS and cultured for a further 24 hours. The media was then changed for DMEM/5% (v/v) FCS/1 mg per mL active G418 (Invitrogen) and the plates incubated for a further 10 days, changing the media every 4-5 days or when a lot of dead cells were present. Once most of the cells were dead/detached, the G418 content of the media was reduced to 0.5 mg/mL and the plates incubated until distinct colonies were present. The colonies were then fixed in 1:3 Acetic acid: Methanol, stained with 0.1% (w/v) Trypan Blue (Gibco-BRL) and counted.

### Marker Rescue Assay

293T cells were plated at 2 × 10^6 ^cells in 4.5 mL in 60 mm dishes and incubated at 37°C/5% CO_2 _for 3 hours, after which the media was replaced with 1 mL of 0.45 μm filtered virus and 4 mL DMEM/5% (v/v) FCS and made 4 μg/mL (final) polybrene (Sigma). The cells were incubated at 37°C/5% CO_2 _for 24 hours before being split into 2 × T75 and cultured for 7 days with subculturing performed when required. The media was collected and titred for residual virus and the transduced 293T cells were plated in 2 × 150 mm dishes at a density of 1.4 × 10^7 ^cells in 30 mL of medium and incubated at 37°C/5% CO_2 _for 24 hours, after which 16.2 μg of pCMVΔRnr and 10.8 μg of pHCMV-g were transfected *via *calcium phosphate co-precipitation to one plate, and 27 μg of pBluescript transfected into the second, which served as a control. Eight hours post transfection, the culture media was changed for 35 mL DMEM/5% FCS prewarmed to 37°C. The media was collected 48 hours post transfection, concentrated *via *ultracentrifugation (Beckman Optima L-100K) in a SW32 rotor at 50,000 g for 90 minutes at 4°C and assayed for virus titre. The rescued virus was also applied to fresh 293T cells and the rescue cycle performed once more. After the second round of rescue, virus was again titred and 19-23 neomycin resistant colonies from each group were isolated using cloning rings, expanded and genomic DNA prepared and assayed to determine the 5' LTR status of the integrated provirus by PCR.

### PCR for 5'LTR status

Genomic DNA was isolated using the Wizard SV genomic DNA Isolation kit (Promega). Two point five μL of gDNA was combined with 1 μg of primer INT19 (5' TGGAAGGGCTAATTCACTC 3'), 1 μg of primer Nar1R (5' CGGGCGCCACTGCTAGAGATTTTCC 3'), 100 μM dNTPs, 1 × Qiagen PCR buffer and 2.5 Units of Hot Star Taq DNA polymerase (Qiagen). The reaction was cycled under the following conditions; 95°C 15 minutes, followed by 35 cycles of 95°C 30 seconds, 60°C 30 seconds, 72°C 30 seconds, followed by 72°C 10 minutes. The PCRs were then examined on a 3% agarose gel. A repaired/full length LTR exhibited a size of 649 bp for pHIV-1SDmSneo and pRK1neo, and 539 bp for pRK1Δ*att*340/184neo, whereas a 5' deleted LTR exhibited a size of 200 for pRK1neo and pRK1Δ*att*340/184neo and 244 bp for pHIV-1SDmSneo.

### RNA analysis

#### RNA isolation

293T cells were plated at a density of 0.625 × 10^6 ^cells in 2 mL per well in a 6 well plate and incubated for 24 hours. Fugene-6 (Roche) was used to transfect 3.3 μg of the appropriate vector combined with 100 ng each of pcDNA3Tat101ml and of pHCMVRevmlwhvpre [37]. Eight hours post transfection, the media was aspirated and replaced with fresh prewarmed media. The cells were cultured for a further 40 hours, harvested, washed in PBS and recovered *via *centrifugation. RNA was isolated using TRIZol (Invitrogen) according to the manufacturer's instructions.

#### Analysis of Spliced Transcripts

Reverse Transcriptase PCR was performed using Thermoscript Reverse Transcriptase PCR system (Invitrogen) following the recommended method, and using five hundred nanograms of RNA and an oligo dT primer. Four microlitres of cDNA was used in a PCR containing 1 μg of primers PBSF (5' GGGCCCCGAACAGGGACTTG 3') and EYR (5' GGGCCCATATGCTTTACTTGTACAGCT 3'), 1 × Qiagen PCR buffer and Hot Star Taq DNA polymerase (Qiagen) under the following conditions; 94°C 45 seconds, 55°C 30 seconds, 72°C 2 minutes for 35 cycles. PCRs were analysed on a 3% agarose gel to determine splicing patterns.

#### Polyadenylation signal readthrough assay

RNA was treated with TURBO DNAse (Ambion) and reverse transcribed using Thermoscript Reverse Transcription Kit (Invitrogen) with random hexamer primers. The cDNA was then assayed using the *eyfp *(forward primer 5' ACGGCCCCGTGCTG 3' probe 5' FAM-CTGCCCGACAACCACT-NFQ 3' and reverse primer 5' AGGGCGGACTGGTAGCT 3') and *eyfp to pBCKS *(forward primer 5' GAGAAGCGCGATCACATGGT 3' probe 5' FAM-CTGCTGGAGTTCGTGACCGCCG-NFQ 3' reverse primer 5' CAGCTGGCACGACAGGTTT 3') TaqMan real time PCR assays. Each reaction contained 1.5 μL cDNA, 1 × TaqMan Universal PCR Master Mix (Applied Biosystems), 0.9 μM of each primer and 0.25 μM of probe in a total volume of 20 μL and was run on an Applied Biosystems 7300 Real Time PCR machine under the following conditions: 50°C for 2 minutes, 95°C for 10 minutes followed by 40 cycles of 95°C for 15 seconds and 60°C for 3 minutes. Transcript copy number was determined using the ΔΔct method. The readthrough efficiency was calculated as the number of transcripts detected by the *eyfp to pBCKS *PCR normalised to the number of transcripts detected by the *eyfp *PCR.

### Statistical Analysis

All results are given as mean ± standard deviation. Chi squared was performed using the calculator available at . All other statistical analyses, where appropriate, were performed using SigmaStat for Windows version 3.0.0. Analysis was performed using Analysis of Variance (ANOVA) coupled with a multiple comparison as specified in the text, t-test or Chi squared.

## Abbreviations

*att*: HIV-1 attachment sequence; bGH: bovine growth hormone (gene); CRS: *cis*-repressive sequence; *eyfp*: enhanced yellow fluorescent protein; gRNA: genomic RNA; LTR: long terminal repeat; ppt: polypurine tract; RT-PCR: real time-PCR; SIN: self inactivating; SV40:simian virus 40; X-SCID: X-linked severe combined immunodeficiency.

## Authors' contributions

RK performed all experimental work and made a major contribution to the conceptualisation and design of the study. DSA was responsible for the broad concept of the study and the sequence analysis of the marker rescue assay PCR products. Both authors contributed to the preparation of the manuscript.
